# Wound Healing Properties of *Jasione montana* Extracts and Their Main Secondary Metabolites

**DOI:** 10.3389/fphar.2022.894233

**Published:** 2022-05-10

**Authors:** Aleksandra Maria Juszczak, Katarzyna Jakimiuk, Robert Czarnomysy, Jakub Władysław Strawa, Marijana Zovko Končić, Krzysztof Bielawski, Michał Tomczyk

**Affiliations:** ^1^ Department of Pharmacognosy, Faculty of Pharmacy with the Division of Laboratory Medicine, Medical University of Białystok, Białystok, Poland; ^2^ Department of Synthesis and Technology of Drugs, Faculty of Pharmacy with the Division of Laboratory Medicine, Medical University of Białystok, Białystok, Poland; ^3^ Department of Pharmacognosy, Faculty of Pharmacy and Biochemistry, University of Zagreb, Zagreb, Croatia

**Keywords:** *Jasione montana*, flavonoids, luteolin derivatives, fibroblasts, wound healing, antioxidant, enzyme inhibition

## Abstract

The effects of different extracts obtained from *Jasione montana* L. (JM1–JM6) and their main metabolites on biological processes during wound healing were evaluated. The effect on wound closure in the scratch test was established, and collagen type I synthesis and anti-inflammatory effects were assessed by flow cytometry in a human dermal fibroblast model (PCS-201-012). Additionally, the antioxidant activity (DPPH and FRAP) and degree of inhibition of elastase participating in the proliferation processes of skin fibroblasts were determined in an *in vitro* model. The extracts and fractions were analyzed using high-performance liquid chromatography–photodiode array detection (HPLC–PDA) to quantitatively characterize their main polyphenolic compounds. The high antioxidant activity of the JM4–JM5 fractions correlated with the content of luteolin and its derivative 7-*O*-glucoside. Luteolin also showed the highest anti-elastase activity with an IC_50_ value of 39.93 ± 1.06 μg/mL, and its substantial content in the JM4 fraction presumably determines its activity (359.03 ± 1.65 μg/mL). At lower concentrations (<50 μg/mL) of all extracts, cell proliferation and migration were significantly stimulated after 24 h of treatment. The stimulation of cell migration was comparable with that of allantoin, which was used as a positive control. However, most of the tested extracts showed limited capacity to affect collagen type I biosynthesis. Moreover, the tested samples exhibited a complex effect on cytokine secretion, and the strongest anti-inflammatory activity through the moderation of IL-1β, IL-6 and IL-8 was observed for JM4 and luteolin. Based on the obtained results of the quantitative analysis, the anti-inflammatory activity of JM4 may be due to the high content of luteolin. In summary, extracts from *J. montana*, which is flavonoid-rich, promote the viability and accelerate the migration of fibroblasts as well as moderate oxidant and inflammatory processes and elastase activity. Hence, they may be potentially useful for topical therapeutic applications to stimulate the wound healing process.

## 1 Introduction

The wound healing process is considered to be a series of dynamic overlapping reactions leading to proliferation, migration, matrix synthesis and contraction, as well as restoration of function and integrity to damaged tissues, resulting in the restoration of anatomic continuity. In general, the wound healing process consists of four distinct but smoothly overlapping phases, including hemostasis, the inflammatory phase, proliferation and remodeling of new tissue. Hemostasis occurs immediately after injury and is a form of protection of the vascular system and bridges invading cells. Transforming growth factor-β (TGF-β), which stimulates fibroblasts, and platelet-derived growth factor (PDGF) and vascular endothelial growth factor (VEGF), which are created by *inter alia* fibroblasts, are involved in promoting post-inflammatory wound healing cascades. In addition to VEGF, other important inflammatory cytokines regulated by tumor necrosis factor-α (TNF-α) are interleukin 6 (IL-6), with both pro- and anti-inflammatory properties, and interleukin 8 (IL-8) with chemotactic properties and downregulation of collagen expression by fibroblasts (Barrientos et al., 2008; Wedler et al., 2014). The proliferative phase, during which the aforementioned cytokines are still involved, is characterized by epithelialization, which leads to cell detachment, proliferation, migration and differentiation. The proliferation stage also includes angiogenesis in newly developed cells and tissue, formation of granulation tissue and deposition of collagen produced by proliferating fibroblasts (Gothai et al., 2016). The most common cells in the dermis particularly involved at each stage of the process are fibroblasts, which generate the proliferative phase of repair and are also involved in phases such as early inflammation and full epithelialization of the damaged tissue (Addis et al., 2020). The healing process is closely linked to the modulation of the secretion of many growth factors, extracellular matrix (ECM) signaling proteins, and cytokines; collagen deposition; and the control of oxidative stress (Bainbridge, 2013). ECM components such as collagen, fibrin, elastin, fibronectin, proteoglycans and others function as significant protagonists of the survival, proliferation and function of fibroblasts. The interaction between the ECM and these cells is a form of autocrine regulation that is essential in wound healing. Furthermore, matrix metalloproteinases (MMPs), such as collagenase and elastase, play an important role in regulating ECM degradation and deposition and thus affect tissue remodeling and repair (Tracy et al., 2016; Jakimiuk et al., 2021).

There is an association between wound healing processes and oxidative stress-regulating activity, and one of the main factors that play a fundamental role in wound healing is oxidation; hence, antioxidant polyphenolic substances of plant origin may be a particularly important aspect (Ustuner et al., 2019; Comino-Sanz et al., 2021; Monika et al., 2022). The presence of several polyphenolic compounds, mainly of a flavonoid nature, was revealed in the aerial parts of *Jasione montana* L. (Campanulaceae). To date, 5,7,3',4'-tetrahydroxyflavone (luteolin) (**22**), luteolin 7-*O*-*β*-D-glucoside (cynaroside) (**12**) and luteolin 7-*O*-*β*-D-xylosyl-(1-2)-*β*-D-glucoside (luteolin 7-*O*-sambubioside) (**9**) have been isolated. Moreover, the anticancer effect of *J. montana* has been documented in an *in vitro* model of skin melanoma (Juszczak et al., 2021). As, from the pharmacological point of view, stimulation of proliferation, migration and differentiation of dermal fibroblasts, as well as anti-inflammatory or antioxidant effects, are critical aspects of the wound healing process, the aim of this study was to evaluate the effects of *J. montana* extracts and their main metabolites, such as luteolin, luteolin 7-*O*-glucoside and luteolin 7-*O*-sambubioside, on processes involved in wound healing.

## 2 Materials and Methods

### 2.1 Plant Material and Preparation of Extracts JM1–JM3 and Fractions JM4–JM6

Aboveground parts of *J. montana* were collected and identified as reported previously by Juszczak and coauthors. The plant material was pretreated and then processed to obtain extracts **JM1**–**JM3** and fractions **JM4**–**JM6** following a previously described procedure (Juszczak et al., 2021).

### 2.2 Quantitative Analysis of Extracts JM1–JM3 and Fractions JM4–JM6 by HPLC–PDA

The quantification of selected secondary metabolites and the sum of their derivatives in extracts (**JM1–JM3**) and fractions (**JM4–JM6**) was performed in compliance with the International Council for Harmonisation of Technical Requirements for Pharmaceuticals for Human Use (ICH) recommendations (Okuda et al., 2014). Details of the optimization and validation high-performance liquid chromatography with a photodiode detector (HPLC-PDA) method are presented in the Supplementary Table S1.

### 2.3 DPPH Radical-Scavenging Assay

The antioxidant activity of the **JM1**–**JM6** extracts and their main compounds (**9**, **12**, and **22**) against 2,2-diphenyl-1-picrylhydrazyl (DPPH) radicals was estimated according to the method previously described (Ciganović et al., 2019) with some modifications. All extracts (**JM1**–**JM6**) and main components of the tested extracts (**9**, **12**, and **22**) were isolated and identified as previously described (Juszczak et al., 2021). A total of 130 μL of sample solution at various concentrations was mixed with 70 μL of DPPH (0.2 mg/mL) in a 96-well plate. Then, the reaction mixture was incubated in the dark at room temperature. After 30 min, the absorbance was recorded at 517 nm using a microplate reader (BioTek Instruments microplate spectrophotometer EPOCH 2, Oxfordshire, United Kingdom). A blank solution was prepared by mixing methanol and DPPH solution. Trolox was used as a positive control. The experiment was performed in triplicate.

RSA(%)=(C-S)/C×100%

where C is the negative control and S is the sample. Concentration of the tested samples, which scavenges 50% of free radicals present in the solution (IC_50_), was calculated.

### 2.4 Ferric Reducing Antioxidant Potential

Determination of the ferric reducing antioxidant potential (FRAP) was performed using a ferric reducing antioxidant power assay kit. Ten microliters of each sample (**JM1**–**JM6**, **9**, **12**, **22**) was mixed with 190 µL of supplied reaction mix (FRAP assay buffer, FeCl_3_ solution, FRAP probe). The absorbance was measured after 1 h of incubation at 37°C at 594 nm using a microplate reader. A blank solution contained 10 µL of MeOH instead of extract/compound solution. For further calculations, the ferrous standard curve was evaluated. The values are given as mM ferrous equivalents. All tests were executed in triplicate.

### 2.5 Elastase Inhibitory Activity

The measurement of elastase inhibition was modified slightly according to a previous method (Wittenauer et al., 2015). The procedure was performed in 100 mM Tris buffer (pH = 8.0). Briefly, 100 µL of extracts (**JM1**–**JM6**) (100–600 μg/mL) or compounds (**9**, **12**, and **22**) (10–200 μg/mL) and 100 µL of Tris buffer were mixed with 25 µL of porcine pancreatic elastase solution (0.05 mg/mL). Then, the mixtures were preincubated at 25°C for 5 min, and 70 µL of AAAPVN (0.4 mg/mL) was added. After another 15 min of incubation, the release of *p*-nitroaniline was monitored at 410 nm using a microplate reader. A blank solution was prepared by using buffer instead of sample solution. Quercetin was employed as a positive control. The calculations were performed as follows:

ElInh(% )=(C-S)/C×100%

where C is the negative control and S is the sample.

### 2.6 Cell Culture

The normal human dermal fibroblast cell line (PCS-201-012) was cultured in DMEM blended with 10% fetal bovine serum (FBS), 10 μg/mL streptomycin, and 10 units/mol penicillin. The cells were cultured in 5% CO_2_ and fully humidified at 37°C. All tested compounds were dissolved at a final vehicle (DMSO) concentration of not more than 0.5% (*v/v*). Cells cultured in drug-free DMEM were used as controls, and cells treated with DMSO alone were used as solvent controls. All extracts (**JM1**–**JM6**) and the main components of the tested extracts (**9**, **12**, and **22**) were analyzed at the following concentrations: 10, 25, 50, 100, 200, and 300 μg/mL.

#### 2.6.1 Proliferation and Migration Assay

The *in vitro* wound healing activity of *J. montana* extracts and isolated compounds was determined by a scratch assay, which is a model of a wound in which monolayer skin cells, including fibroblasts, react to the disruption of contact between cells and stimulate proliferation and migration by modulating the concentration of growth factors and cytokines at the edge wounds (Liang et al., 2007). For this purpose, fibroblast cells were seeded at a density of 2.5 × 105 in 6-well cell culture dishes and incubated for 24 h at 37°C. After incubation, a linear scratch was made with a universal sterile 200 µL pipette tip. Cells were incubated with the tested extracts (**JM1**–**JM6**) and compounds (**9**, **12**, and **22**) at concentrations of 10, 25, 50, 100, 200 and 300 μg/mL for 24 h at 37°C. Allantoin, a plant-derived commercialized drug, was used as the positive control at a concentration of 50 μg/mL to judge the rate of cell migration (Sarkhail et al., 2020). After incubation, the migrated cells were washed twice with PBS and observed under a phase contrast microscope. The progress of migration, proliferation and closing of the wound before and after treatment with the tested samples was monitored by imaging with a phase contrast microscope (Nikon Eclipse Ti, Tokyo, Japan) at × 100 magnification and NIS-Elements 3.0 imaging software (Nikon Instruments Inc., Melville, NY, United States).

#### 2.6.2 Biosynthesis of Collagen Type I

The effect of the tested extracts and fractions (**JM1**–**JM6**), as well as their main compounds (**9**, **12**, and **22**) on collagen type I biosynthesis was determined using a method of cytometric staining of the surface anti-collagen type I according to the manufacturer’s protocols. Fibroblasts were incubated for 24 h at 37°C with the test samples at concentrations of 10, 25, 50, 100, 200, and 300 μg/mL. Allantoin (50 μg/mL) was used as a positive control. After incubation, fibroblasts were washed with PBS, trypsinized, resuspended in DMEM and centrifuged (1,200 rpm, 10 min, 4°C). The supernatant was removed, and the cells were washed with 2 mL of stain buffer. Then, the cells were incubated for 30 min on ice with 100 µL of stain buffer and 1 µL of antibody. After complete incubation, 2 mL of stain buffer was added. After consecutive centrifugation (1,200 rpm, 10 min, 4°C), the supernatant was removed, the cells were resuspended in 300 µL of buffer and then immediately subjected to analysis on a flow cytometer (BD FACSCanto II flow cytometer, San Jose, CA, United States) calibrated with BD Cytometer Setup and Tracking beads (BD Biosciences, San Diego, CA, United States). The median fluorescence intensity was calculated using FACSDiva software (BD Biosciences Systems, San Jose, CA, United States) and analyzed by FCS Express 7 software (DeNovo Software, Pasadena, CA, United States).

#### 2.6.3 Flow Cytometric Analysis of Inflammatory Cytokines

The effect of the tested extracts and fractions (**JM1**–**JM6**) as well as **9**, **12**, and **22** on cytokine (IL-1β, IL-6 IL-8, IL-10, IL-12p70 and TNF) secretion was determined using the Cytometric Bead Array (CBA) Human Inflammatory Cytokine Kit according to the manufacturer’s protocols. After 24 h of incubation with the test samples at concentrations of 10, 25, 50, 100, 200, and 300 μg/mL, fibroblast cells were washed with PBS, trypsinized, resuspended in DMEM and centrifuged (1,200 rpm, 10 min, 4°C). The supernatant was then replaced with 50 μL of assay diluent with the addition of 50 μL of assay beads and 50 μL of PE-labeled antibodies (detection reagent) from the human inflammatory cytokine kit. Incubation was performed for 3 h at room temperature without daylight, after which the fibroblast cells were washed and centrifuged (1,200 rpm, 5 min, 4°C). The cell pellet was resuspended in 300 μL of wash buffer and then immediately subjected to analysis by FCAP Array v3 software on a BD FACSCanto II flow cytometer (both from BD Biosciences Systems, San Jose, CA, United States) calibrated with BD Cytometer Setup and Tracking beads (BD Biosciences, San Diego, CA, United States).

### 2.7 Statistical Analysis

All numerical data are expressed as the mean ± standard deviation (SD) from at least three independent repeats. GraphPad Prisma eight software (GraphPad Software, San Diego, CA, United States) was used for statistical analysis. Statistical differences were assessed using one-way ANOVA followed by Dunnett’s multiple comparisons test. Statistically significant values were considered under the condition of *p* < 0.05. MS Excel 2019 software with the Data Analysis add-on was used for statistical analysis and determination of linear regression parameters. The parameters were obtained using ANOVA with a confidence level of 95%.

## 3 Results

### 3.1 Quantitative Analysis of Extracts JM1–JM3 and Fractions JM4–JM6 by HPLC–PDA

The main components of the aerial parts of *J. montana* were successfully identified by LC–MS analysis in a previous report (Juszczak et al., 2021). The extracts from *J. montana* were quantified by HPLC-PDA by preparing the calibration curves of the four reference substances. Table 1 presents the contents of major components, luteolin 7-*O*-sambubioside (**9**), luteolin 7-*O*-glucoside (**12**), and luteolin (**22**), as well as their derivatives in the extracts (**JM1–JM3**) and the fractions (**JM4–JM6**). Each investigated extract has variations in quantitative content with regard to the main compounds. The phytochemical composition of **JM1**–**JM6** is relatively limited, however, the dominant compounds in the studied extracts are present in significant amounts. The dominant compounds present in **JM4** are **22** (72.13 ± 3.86 mg/g dry fraction) and **12** (40.21 ± 0.85 mg/g dry fraction), while **JM5** contains a significant amount of **12** (383.16 ± 1.36 mg/g dry fraction), and other flavonoid derivatives. The high content of dominant constituents in these fractions was the basis for the attempt to isolation process of compounds **9**, **12**, and **22** (Juszczak et al., 2021).

**TABLE 1:** Quantification of major compounds (compounds **9**, **12** and **22**) in *J. montana* extracts (**JM1**–**JM3**) and fractions (**JM4**–**JM6**) by HPLC–PDA.

Compounds	JM1	JM2	JM3	JM4	JM5	JM6
	mg/g dry Extract/fraction					
**9** ^b^	10.30 ± 0.03	6.57 ± 0.03	BLQ	nd	BLQ	6.77 ± 0.08
**12**	52.02 ± 0.17	31.21 ± 0.07	11.10 ± 0.08	40.21 ± 0.85	383.16 ± 1.36	nd
**22**	5.78 ± 0.11	7.82 ± 0.02	8.18 ± 0.09	72.13 ± 3.86	nd	nd
luteolin derivatives^a^ ^,^ ^b^	83.66 ± 0.28	53.81 ± 0.14	23.11 ± 0.02	53.51 ± 1	474.87 ± 1.86	20.76 ± 0.13

**9** – luteolin 7-*O*-sambubioside; **12** – luteolin 7-*O*-*β*-D-glucoside; **22** – luteolin.

a Excluding luteolin.

b Expressed as equivalent of luteolin 7-*O*-glucoside with standard deviation; BLQ, below the limit of qualification; nd, not detected.

### 3.2 Antioxidant Activity of *J. montana* Extracts and Isolated Compounds

The effects of *J. montana* extracts and isolated compounds on antioxidant activity are given in Table 2. The analysis of antioxidant potential by the DPPH and FRAP methods confirmed that the highest activity for extracts and fractions are connected with increasing polyphenolic compound content (Sulaiman et al., 2013). Based on the results obtained, the tested plant had high antioxidant activity in the presence of compounds **22** (DPPH: IC_50_ = 10.6 ± 0.7 μg/mL, FRAP: 60.59 ± 0.5 mM Fe^2+^/mL) and **12** (DPPH: IC_50_ = 16.6 ± 0.7 μg/mL, FRAP: 51.9 ± 0.5 mM Fe^2+^/mL). As stated by the quantitative HPLC analysis, the highest contents of **22** and **12** were prevalent in the **JM4** and **JM5** fractions, which is consistent with their free radical-scavenging potential, with IC_50_ values of 72.7 ± 2.3 and 42.7 ± 1.7 mM Fe^2+^/mL and reducing powers of 43.87 ± 1.32 and 51.55 ± 1.65 mM Fe^2+^/mL, respectively. Moreover, all tested compounds (**9**, **12**, and **22**) possessed stronger antiradical potential in the DPPH method than a positive control, Trolox (IC_50_ = 58.6 ± 0.1 μg/mL).

**TABLE 2:** Antiradical activity against DPPH radical (IC_50_, µg/mL) of **JM1**–**JM6** extracts and compounds (**9**, **12**, and **22**), FRAP values (mM Fe^2+^ per mL).

Sample	DPPH, IC_50_ (µg/mL)^a^	FRAP (mM Fe^2+^eq/mL)^b^
**JM1**	457.15 ± 5.2	34.33 ± 0.99
**JM2**	276.60 ± 3.8	46.53 ± 0.87
**JM3**	261.18 ± 4.2	41.31 ± 0.99
**JM4**	72.69 ± 2.3	43.87 ± 1.32
**JM5**	42.69 ± 1.7	51.55 ± 1.65
**JM6**	848.47 ± 4.4	10.63 ± 0.92
**Compound 9**	20.34 ± 0.8	60.59 ± 0.46
**Compound 12**	16.59 ± 0.7	51.88 ± 0.53
**Compound 22**	10.65 ± 0.7	51.81 ± 0.78

a All data are represented as the mean IC_50_ values.

b Ability to reduce the Fe^3+^ complex to ferrous Fe^2+^.

### 3.3 Elastase Inhibition Activity of *J. montana* Extracts

Inhibition of elastase activity for crude extracts from *J. montana* was performed. The effect of their anti-elastase potential expressed as IC_50_ values is summarized in Table 3. Among all tested samples, only three (**JM4**, **JM5**, and **22**) exhibited moderate anti-elastase effects. Compound **22** (IC_50_ = 39.93 ± 1.06 μg/mL) showed the highest activity, followed by **JM4 (**IC_50_ = 359.03 ± 1.65 μg/mL) and **JM5** (IC_50_ = 385.03 μg/mL). On the other hand, **JM1**–**JM3**, **JM6**, **9** and **12** showed no significant inhibitory activity.

**TABLE 3:** Elastase inhibitory activity of **JM1**–**JM6** extracts; compounds **9**, **12**, and **22**; and quercetin expressed as IC_50_ (µg/mL).

Sample	IC_50_ (µg/mL)
**JM1**	na
**JM2**	na
**JM3**	na
**JM4**	359.03 ± 1.65
**JM5**	385.03 ± 1.87
**JM6**	na
**Compound 9**	na
**Compound 12**	na
**Compound 22**	39.93 ± 1.06
**Quercetin**	22.36 ± 0.86

na, not active; PC, positive control (quercetin).

### 3.4 Scratch Wound Healing of Fibroblast Cells by the Influence of *J. montana* Extracts

**FIGURE 1:**
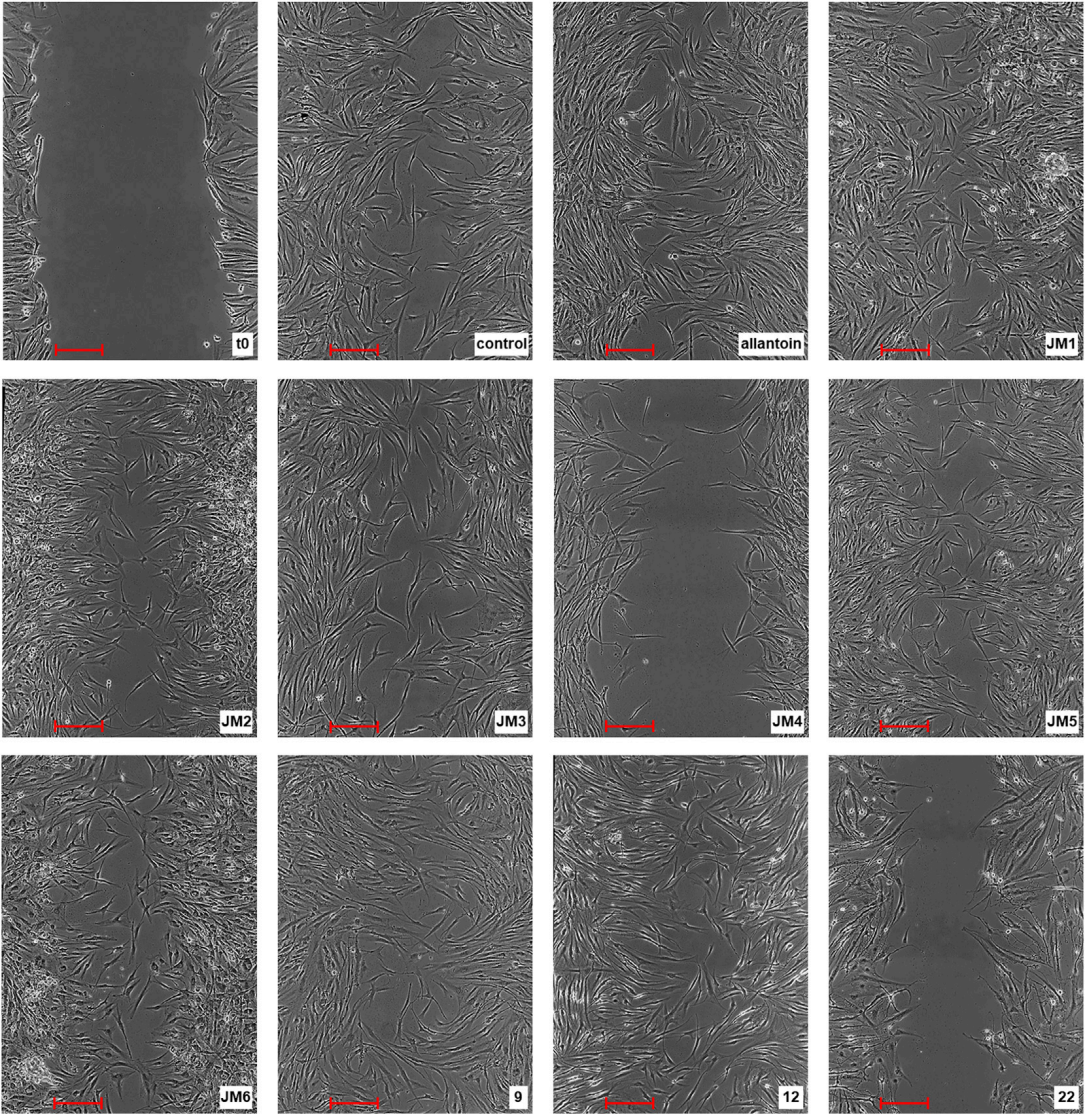
Representative image of the *in vitro* scratch migration assay in fibroblast cells immediately after the wounding (t0), untreated (control) or treated with allantoin (50 μg/mL), extracts **JM1**–**JM6** (50 μg/mL), or compounds **9**, **12** (25 μg/mL) or **22** (10 μg/mL) after 24 h incubation evaluated by phase contrast microscopy (magnification × 100) (scale bar 250 µm).

To assess the *in vitro* wound healing effect of *J. montana*, fibroblast migration concerning the closure of the uncovered scratched area by the scratch assay was monitored. Data obtained from an *in vitro* scratch model using fibroblast cells showed that *J. montana* extracts and isolated compounds significantly enhanced fibroblast cell migration toward an induced temporary interruption at most of the tested concentrations to varying extents compared with the migration of cells into the wounded area incubated in medium free from tested samples 24 h after wounding (Figure 1). Wound closure was stimulated in a concentration range of 10–300 μg/mL, with the tested fractions, extracts and compounds being less effective at higher concentrations. For the **JM1**–**JM3** extracts, **JM5**–**JM6** fractions and compounds **9** and **12**, the cell migration into the injured area was significantly increased up to a concentration of 100 μg/mL. However, above this concentration, significantly less stimulation of fibroblasts, as well as a reduction in cell adhesion and loss of intercellular connections, was observed (data not shown). **JM1** and **9** (50 μg/mL) proved to be most effective in the scratch test, and cell migration to the wound area after 24 h of incubation was comparable with the healing-promoting activity visualized for allantoin (50 μg/mL). Both treatments returned cells to a confluent or near confluent state within 24 h, comparable with the untreated control cells. In contrast, a distinct trend was observed for **22** and **JM4**, which were capable of stimulating cells only at a low concentration (10 μg/mL). This observation coincides with the higher cytotoxicity of these samples presented in the previous report (Juszczak et al., 2021).

### 3.5 Collagen Type I Biosynthesis

**FIGURE 2:**
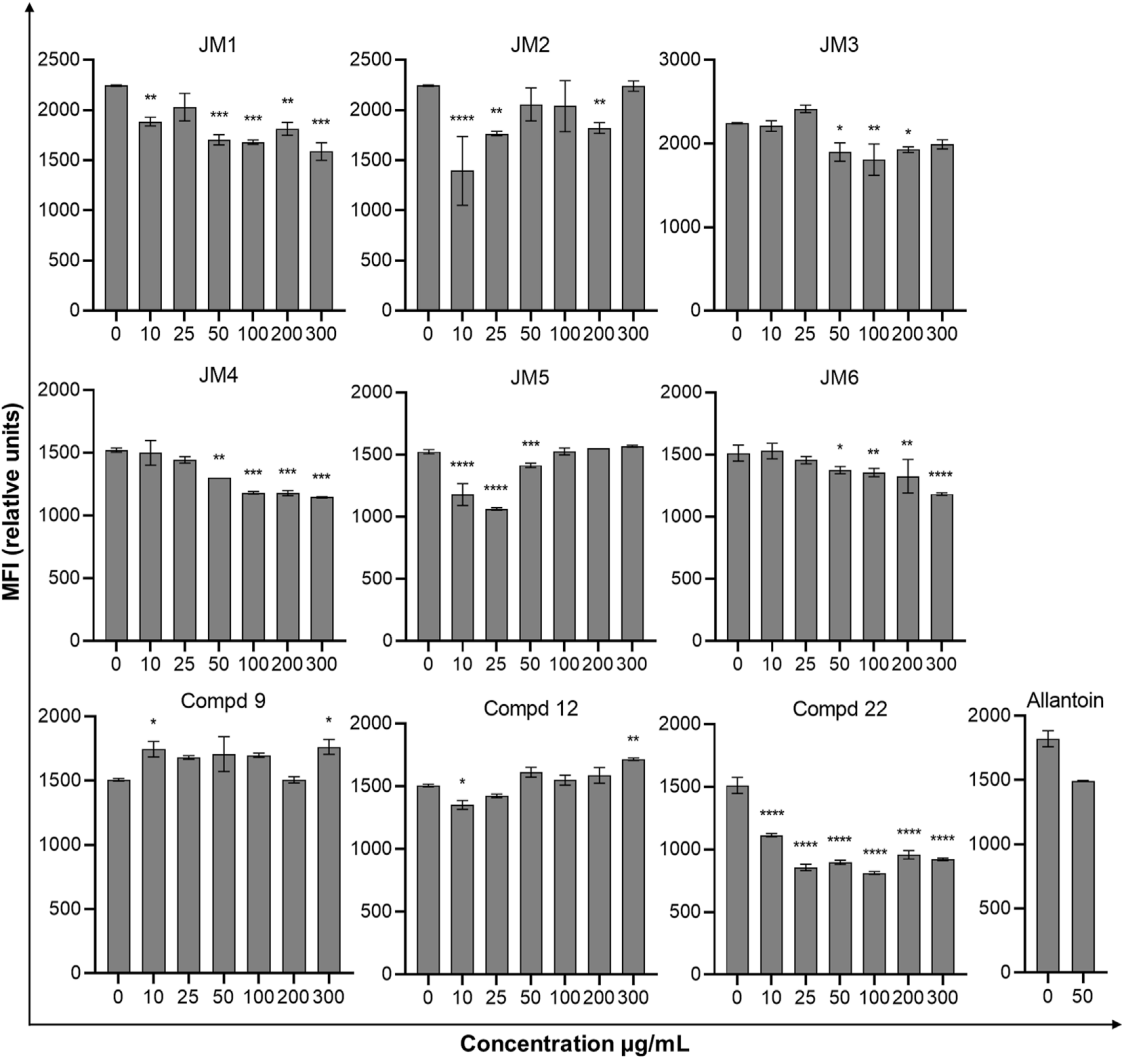
Effect of *J. montana* extracts (**JM1**–**JM6**) and their main compounds (**9**, **12**, and **22**) (10–300 μg/mL) and allantoin (50 μg/mL) on the collagen type I content in fibroblast cells. Data are expressed as the percentage of the median fluorescence intensity compared with the control. Data are presented as the median fluorescence intensity (MFI) from three independent experiments (*n* = 3) performed in duplicate. *p < 0.05 versus the control group, **p < 0.01 versus the control group, ***p < 0.001 versus the control group, ****p < 0.0001 versus the control group.

In the further evaluation of the wound healing properties, we examined the intracellular expression of the anti-collagen type I antibody fluorescein conjugation specific for collagen type I under the influence of *J. montana* extracts and their main compounds in fibroblast cells. The results are expressed as the median fluorescence intensity (MFI) and are shown in Figure 2 and Supplementary Figure S1. Most of the tested extracts showed limited capacity to affect collagen biosynthesis. After treatment with **JM3** at a concentration of 25 μg/mL, only a slight increase in collagen content was observed in fibroblast cells (MFI: 2,417 ± 45.2 vs. 2,244 ± 7.1 control). The observed effect was not dose dependent. The pure isolated compounds showed higher activity than the extracts. Compounds **9** and **12** at the highest tested concentrations significantly increased the soluble collagen content in fibroblast cells compared with untreated control cells. The highest expression of collagen antigen was observed after treatment with **9** at a concentration of 300 μg/mL (MFI: 1763 ± 58 vs. 1,507 ± 9.2 control). Slightly weaker expression was recorded after incubation with **12**, achieving a stimulatory effect on collagen production at a concentration of 300 μg/mL (MFI: 1717 ± 10.6 vs 1,507 ± 9.2 control). Comparing the results obtained for the reference sample, allantoin, it is clear that most of the tested samples had higher activity than the positive control. Moreover, allantoin at a concentration of 50 μg/mL induced downregulation of collagen antigen expression in fibroblast cells (MFI: 1,494 ± 2.8 vs. 1822 ± 62.2 control).

### 3.6 Effect of *J. montana* Extracts on Cytokine Secretion

**FIGURE 3:**
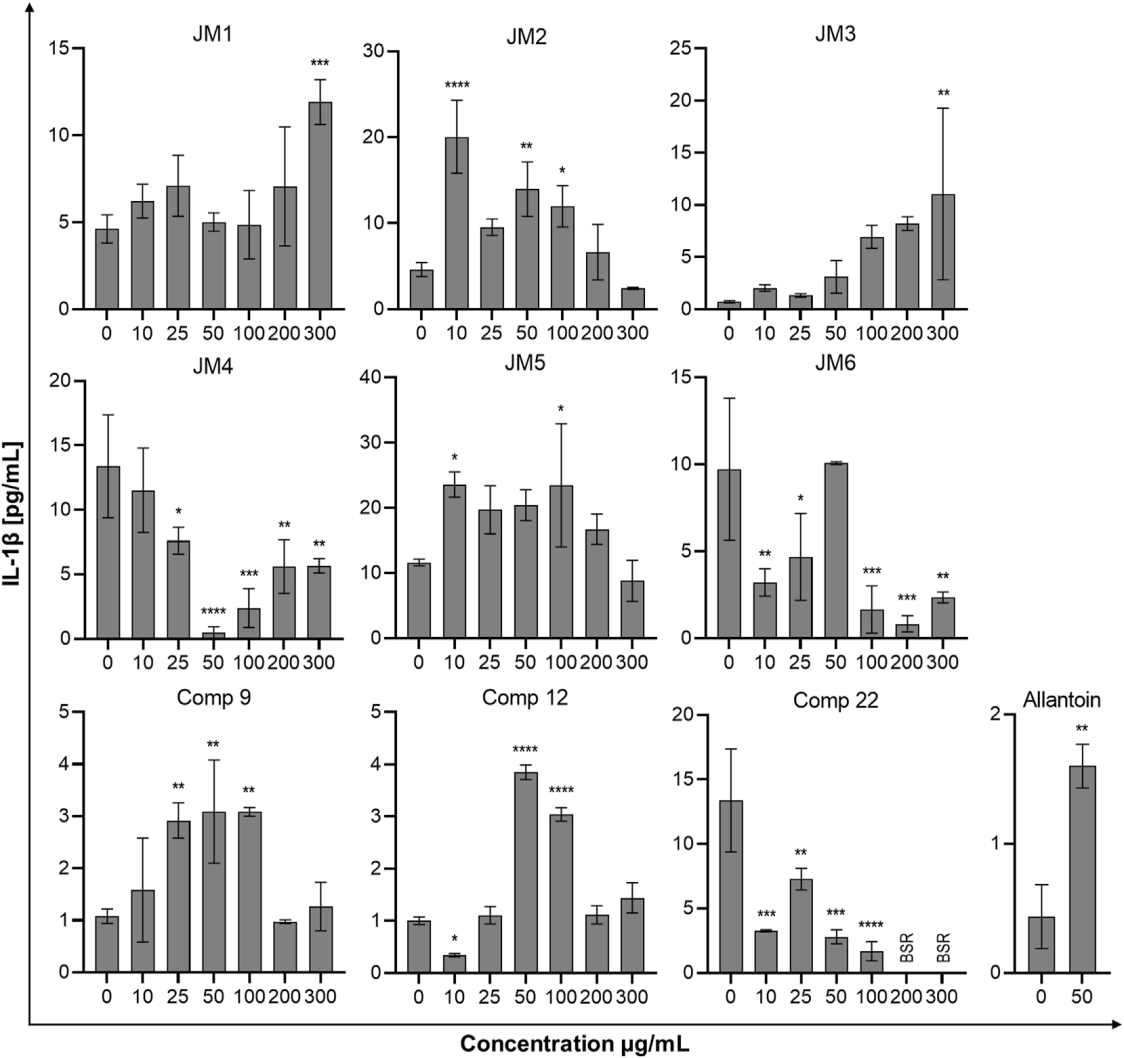
Effects of the *J. montana* extracts **JM1**–**JM6** and their main components (**9**, **12**, and **22**) (10–300 μg/mL) and allantoin (50 μg/mL) on IL-1β production inhibition. Mean values from three independent experiments (n = 3) performed in duplicate are presented. *p < 0.05 versus the control group, **p < 0.01 versus the control group, ***p < 0.001 versus the control group, ****p < 0.0001 versus the control group. BSR, below the standard range (values lower than the detection limit).

**FIGURE 4:**
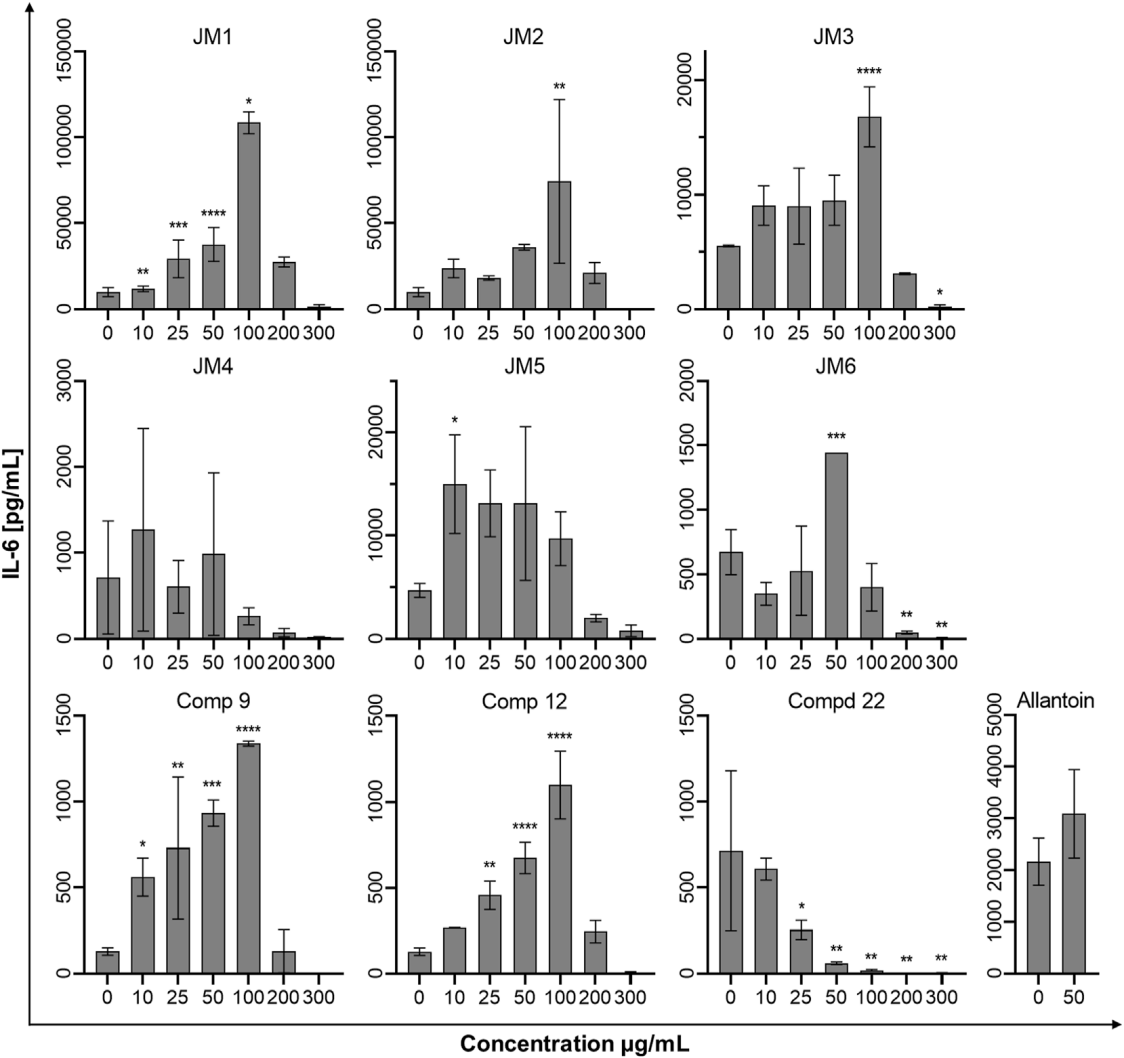
Effects of the *J. montana* extracts **JM1**–**JM6** and their main components (**9**, **12**, and **22**) (10–300 μg/mL) and allantoin (50 μg/mL) on the release of IL-6. Mean values from three independent experiments (*n* = 3) performed in duplicate are presented. *p < 0.05 versus the control group, **p < 0.01 versus the control group, ***p < 0.001 versus the control group, ****p < 0.0001 versus the control group.

**FIGURE 5:**
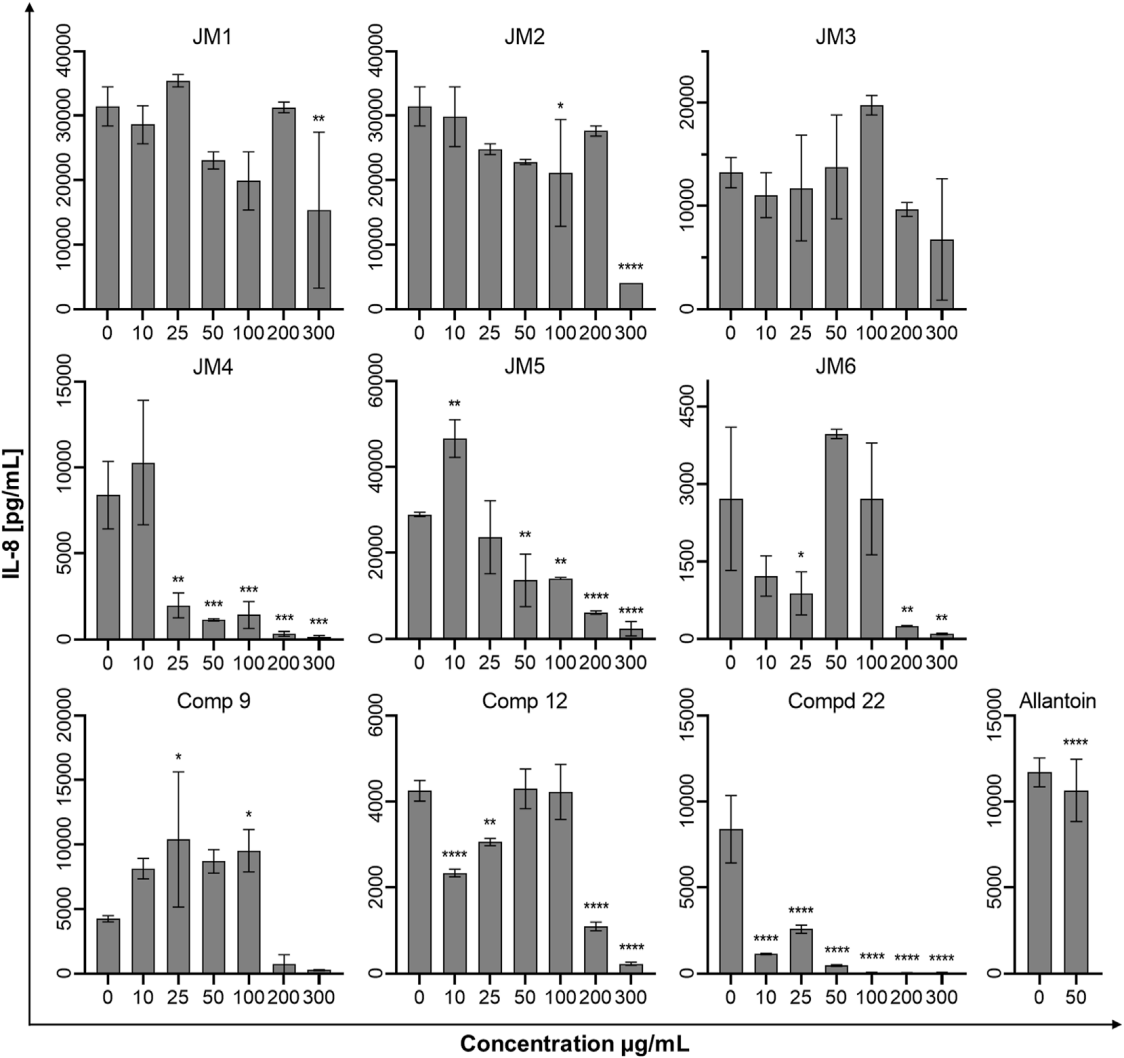
Effects of the *J. montana* extracts **JM1**–**JM6** and their main components (**9**, **12**, and **22**) (10–300 μg/mL) and allantoin (50 μg/mL) on IL-8 production inhibition. Mean values from three independent experiments (*n* = 3) performed in duplicate are presented. *p < 0.05 versus the control group, **p < 0.01 versus the control group, ***p < 0.001 versus the control group, ****p < 0.0001 versus the control group.

To assess the immune status of human fibroblast cells in response to *J. montana* extracts and their major compounds, the levels of representative cytokines in cellular supernatants were examined by flow cytometry. All tested samples showed a complex effect on cytokine secretion that depended on the cytokine considered as well as the concentration of the samples tested. As shown in Figures 3, 5, the expression levels of interleukin 1β (IL-1β) and IL-8 gradually decreased for some extracts and compounds. The highest decrease in the secretion of the labeled pro-inflammatory cytokine IL-1β occurred for all tested concentrations of the **JM4** fraction (10–300 μg/mL) and **22.** After incubation with 50 μg/mL of **JM4**, a decrease in IL-1β to 0.5 ± 0.4 pg/mL vs 13.4 ± 5.6 pg/mL control, and under the influence of 100 μg/mL of **22** 1.7 ± 1.1 pg/mL vs 13.4 ± 5.6 pg/mL control. At the same time, at concentrations of 200–300 μg/mL of **22**, a strong reduction in IL-1β expression below the sensitivity of the method was observed. Furthermore, at higher concentrations (100–300 μg/mL), the **JM2** extract and **JM5** and **JM6** fractions showed a significant reduction in IL-1β release. In the case of the proinflammatory IL-8, a decrease in its expression was most pronounced after treatment with **JM2**, **JM4, JM5**, and **22**. The **JM4** fraction and **22** (300 μg/mL) appeared again to be the most significant inhibitors of proinflammatory cytokine expression (154 ± 102.8 pg/mL vs 8,396.8 ± 2,771.1 pg/mL control and 60.8 ± 8 pg/mL vs 8,396.8 ± 2,771.1 pg/mL, respectively). Based on the quantitative analysis results, the anti-inflammatory activity of **JM4** may be due to the high content of **22** (Table 1). Nevertheless, the other extracts mentioned above showed anti-inflammatory activity in a dose-dependent manner. Moreover, extracts such as **JM1**, **JM3**, **JM6**, and compounds **9** and **12** resulted in reduced IL-8 levels in the higher ranges of tested concentrations (100–300 μg/mL). However, there was a significant increase in the aforementioned proinflammatory cytokine levels after treatment with lower concentrations of **9** (10–100 μg/mL). The highest increase in IL-6 secretion occurred for **JM1** (100 μg/mL), **JM2** (100 μg/mL), **JM3** (100 μg/mL), **JM5** (10 μg/mL), **JM6** (50 μg/mL), **9** and **12** (100 μg/mL) (Figure 4). However, at higher concentrations (300 μg/mL) of the tested samples, there was a drastic decrease in the level of this cytokine. **JM4** and **22** induced a dose-dependent decrease in IL-6 expression levels below that of the untreated control. Compared with the results obtained for the reference sample, allantoin, a decrease in IL-8 expression and upregulation of IL-6 can be observed, but the effect was weaker than for the most active samples tested. However, a significant effect of allantoin on the stimulation of proinflammatory IL-1β levels was observed. Cytokine values lower than the detection limit (IL-10, IL-12p70, and TNF) were considered undetectable and are not shown. The most pronounced effects were observed for the most highly expressed cytokines.

## 4 Discussion

Repair of disrupted skin structure is a complex process leading to the restoration of tissue function in a series of overlapping phases (Soib et al., 2020). Changes at each stage of normal wound healing can result in a delay or inability to repair the disrupted structure (Süntar et al., 2012). In the case of diseases such as obesity and diabetes, the incidences of which are increasing rapidly, patients are at a high risk of developing chronic wounds due to the delay, impairment and uncoordinated healing process (Muniandy et al., 2018). Moreover, impaired repair processes, such as skin and underlying tissue injuries, are encountered in cardiovascular diseases and disorders during cancer therapy (Martinengo et al., 2019). Therefore, to restore the regularity of the wound healing process, the use of substances capable of accelerating the restoration of the physical barrier, as well as protection against unfavorable factors causing wound pathology, may be necessary. The role of certain medicinal plants and phytoconstituents in the wound healing process may represent an attractive approach to their therapeutic properties (Marume et al., 2017; Ustuner et al., 2019). Moreover, the World Health Organization (WHO) defines recommendations for the use of medicinal plant treatment due to their safety and efficacy (World Health Organization, 2013). Previous reports identify *J. montana* as a raw material rich in polyphenolic substances, mainly flavonoids (Juszczak et al., 2021), which are well known for their significant antioxidant properties. Hence, the potential of extracts and fractions from *J. montana* in stimulating wound healing, as well as various biological activities, such as antioxidant and anti-inflammatory properties, was investigated.

The need to measure antioxidant potential is well-reported; such measurements are conducted for significant comparison of food or cosmeceutical products for delivery of quality determination for regulatory cases and health-promoting issues. Overproduction of reactive oxygen species (ROS) caused by skin damage can induce apoptotic changes, damage living tissues, delay or completely stop the healing process due to damage to cell membranes and destruction of proteins, lipids and ECM elements (Ustuner et al., 2019). In the current work, the antioxidant properties of *J. montana* and its main components were established using two different chemical methods, including the DPPH free radical scavenging and FRAP assay. In the DPPH assay, **9** (luteolin 7-*O*-sambubioside), **12** (luteolin 7-*O*-glucoside), **22** (luteolin) and the fractions with their highest content (Table 1), **JM4** and **JM5**, exhibited the strongest ability to scavenge the radicals. Similar findings were revealed in the FRAP method. The best reducing abilities were found in **JM5**, followed by **JM4,** while the lowest activity was observed in **JM6** in both the FRAP and DPPH assays. In accordance with the assays performed, the best antioxidant abilities were found in **JM4**, **JM5** and compounds **9**, **12**, and **22**. The observations could clearly be explained by the higher concentration of the total phenolic compounds in the fractions and extracts. These conclusions are supported by previously reported studies and available literature data (Mainka et al., 2021).

Fibroblasts, alongside keratinocytes, are the predominant cells in the wound closure mechanism and constitute a major target in the commercial design of therapeutic preparations (Ustuner et al., 2019). Their proliferation and migration to the wound site play a key role during the re-epithelialization process of restoring skin integrity, generating new granulation dermis and new collagen structures to support other cells (Bainbridge, 2013). The strength of both of these proliferation phase mechanisms has been assessed using the preferred wound healing model of the *in vitro* scratch test, a useful method for mimicking cell migration during wound closure *in vivo* (Liang et al., 2007). The present study shows that *J. montana* extracts and their major metabolites can promote wound closure and granulation tissue formation by inducing fibroblast cell migration in the scratch assay, a critical step in the proliferation phase, and they simultaneously lack cytotoxic effects as established in a previous study (Juszczak et al., 2021). Furthermore, among the tested samples, **9** and **JM1** most strongly stimulated fibroblast growth and migration (Figure 1). A similar stimulating effect was observed for the **JM5** fraction and its main compound, **12**, probably determining its effects of action (Table 1). The stimulation of fibroblast migration has been repeatedly linked with antioxidant properties (Comino-Sanz et al., 2021); however, only the **JM5** fraction and compounds **9** and **12** partially support the initial hypothesis. Interestingly, fraction **JM4** and its main component probably responsible for its action, **22** (Table 1), show toxicity at the higher concentrations tested despite their high antioxidant properties (Juszczak et al., 2021) as well as moderate fibroblast migration to the wound at lower concentrations. The activity of the *Lavandula stoechas* extract containing luteolin derivatives (54.49 ± 5.02 mg/L), demonstrated by Addis and coauthors, exhibited slight stimulation of the migration and proliferation of fibroblast activity that decreased with increasing incubation time and tested concentration (Addis et al., 2020). On the other hand, Bayrami and coauthors reported a lack of toxicity of luteolin, which was more effective than *Tragopogon graminifolius* extract rich in this flavone, on stimulating cell proliferation in the 3-(4,5-dimethylthiazol-2-yl)-2,5-diphenyl-2H-tetrazolium bromide (MTT) assay and migration in the scratch assay. Moreover, luteolin-induced cell accumulation in the S-phase of the cell cycle was demonstrated, confirming its role in cell proliferation (Bayrami et al., 2018). However, the authors tested luteolin at a concentration of 0.1 μg/mL, which was many times lower than the concentration used in the present study. At the same time, the luteolin-rich fraction from *T. graminifolius* was tested at a concentration of 50 μg/mL, which can be assumed to be in agreement with our reports on the stimulating effect of **JM4** at lower concentrations. Chen et al. (2021) demonstrated that systemic administration of luteolin promotes re-epithelialization of wounds with a well-organized arrangement of fibroblasts. Furthermore, the activity of *Calendula officinalis* extracts, of which luteolin and their derivatives are among the flavonoid components, was reported to stimulate fibroblasts to overgrow the wound (Fronza et al., 2009; Srivastava et al., 2010; Wedler et al., 2014). Nevertheless, according to our data, at higher concentrations, the **JM4** fraction and **22** confer antiproliferative and cytotoxic effects. In both cases, it is probably caused by the high concentration of **22** (Table 1). Moreover, our data about the activity of another predominant constituent, **12**, are in good agreement with those confirmed by the findings of Ustner and coauthors on a *Thymus sipyleus* extract with a significant cynaroside content (0.46 ± 0.01 mg/100 mg extract). The authors simultaneously showed stimulation of proliferation under the influence of the mentioned extracts, as determined by the MTT assay (Ustuner et al., 2019). On the other hand, Karatoprak and coauthors proved that the butanol fraction from *Alchemilla mollis* with a high content of luteolin 7-*O*-glucoside (23.04 ± 0.22 mg/g extract) has at the same time a strong antioxidant potential and toxic activity on L929 fibroblast cells in the sulforhodamine B (SRB) assay. The authors suggest antioxidant as well as toxic effects of luteolin 7-*O*-glucoside (Karatoprak et al., 2017). However, according to Juszczak et al. (2021) and coauthors, this derivative did not show strong cytotoxicity against dermal fibroblast cells and additionally stimulated their migration in the scratch assay (Figure 1).

According to previously performed LC–MS analysis, *J. montana* extracts are present rich in polyphenolic compounds (Juszczak et al., 2021), which have been repeatedly proven to modulate processes closely related to wound healing. In addition to their antioxidant properties, which are associated with repair processes, *p*-coumaric acid as well as apigenin are able to stimulate migration and differentiation of fibroblasts in an *in vitro* and *in vivo* models. Moreover, the anti-inflammatory potential of apigenin through inhibition of TNF-α, IL-6 and IL-1β has been demonstrated (Carvalho et al., 2021; Melguizo-Rodríguez et al., 2021). However, taking into account the complex plant matrix and various phytochemical compositions as well as the fact that the major compounds which are luteolin and its derivatives, present in significant amounts, it cannot be unequivocally stated that other compounds determined in the tested extracts displayed influence on the observed biological effect of *J. montana* extracts or fractions.

From a clinical perspective, the deposition in the wound of collagen produced by fibroblasts, the main protein of the ECM, may be the most important phase of healing and ultimately contributes to the strengthening of the matrix (Lodhi and Singhai, 2013; Tracy et al., 2016). The effect of flavonoid compounds on the stimulation of collagen synthesis in skin fibroblast cultures has already been reported (Ibrahim et al., 2018; Shedoeva et al., 2019). Hence, the effect of *J. montana* extracts on the content of collagen type I in an *in vitro* model of human skin fibroblasts was investigated. However, the observed activity of stimulating collagen type I synthesis for most of the tested samples was limited. The highest activity was observed for compounds **9** and **12**, while, as already mentioned, they did not show the ability to reduce cell viability. In addition, the **JM3** extract slightly increased the content of soluble collagen in the supernatant at a dose of 25 μg/mL. However, it was not a dose-dependent effect; therefore, it is difficult to state which component is responsible for its activity, **12**, **9** or the synergy of the actions of both tested luteolin derivatives (Table 1). Previous reports confirm the hypothesized activity attributed to luteolin glucoside, the presence of which has been reported in extracts from *T. sipyleus*, to alter hydroxyproline levels used as a biomarker for collagen content determinations (Ustuner et al., 2019). However, the effect of stimulating collagen synthesis decreased with increasing concentrations of the administered *T. sipyleus* extract, unlike the results shown in the present study (**12** and **JM5** activity). This fact can be explained by the presence of a second unidentified dominant compound in the mentioned extracts from *T. siplyeus*. Increased content of cross-linked collagen in wounds was also observed by hydroxyproline determination in an *in vivo* model of diabetic mice after topical administration of *Martynia annua* and *Tephrosia purpurea* extracts containing luteolin and was confirmed by histopathological studies. This confirms that flavonoids can lead to the stimulation of collagen synthesis and probably participate in the formation of cross-links as collagen matures (Lodhi et al., 2016). However, our study showed a decrease in collagen content in fibroblast cells after the lowest dose (10 μg/mL) of **22** (luteolin).

Impairment of wound healing leads, *inter alia*, to the overproduction of MMPs and, consequently, to the degradation of the ECM, such as collagen or elastin, and the inhibition of skin re-epithelialization (Wu et al., 2016; Chen et al., 2021). Under physiological conditions, elastin fibers, which contribute to the maintenance of skin elasticity and flexibility, intertwine with the rigid collagen fibers of the ECM. In addition to its important structural role, elastin also has a beneficial effect on wound healing and regeneration (Tracy et al., 2016). In this study, two extracts (**JM4** and **JM5**) and one compound (**22**) were most active elastase inhibitors. However, **JM1**–**JM3**, **JM6**, and compounds **9** and **12** were not active on elastase. When all results were evaluated together, the obtained enzyme inhibitory results may be linked to the chemical composition of the tested extracts and the chemical structure of the compounds. The contradictory results were also observed in the literature, and these phenomena could be explained by the complex nature and possible interactions of phytochemicals in **JM1**–**JM6** (Table 1), as well as *O*-glycosylation at position C7 of the A-ring of the luteolin structure in **9** and **12** (Jakimiuk et al., 2021). Following that, the noticed enzyme inhibitory abilities of **JM4** and **JM5** could be related to the presence of **22**.

The mechanism of chronic wound healing, in addition to the overproduction of MMPs, is also associated with the activation of the prolonged immune response. Inflammation is part of the normal wound healing process; however, in the absence of effective decontamination, this condition can be prolonged with elevated levels of proinflammatory cytokines such as TNF-α and its dependent IL-6 and chemokine IL-8 as well as cytokine IL-1β, impeding the healing itself by limiting proliferation, skin cell differentiation and collagen deposition at the wound site (Ibrahim et al., 2018; Sarkhail et al., 2020). TNF-α and IL-1β levels are elevated in chronic wounds and have a similar response inhibiting ECM synthesis while synergistically increasing MMP production (Barrientos et al., 2008). The modulation of inflammation of the wound healing process may occur through the reduction of pro-inflammatory mediators IL-6 and IL-8 induced by TNF-α secreted from activated fibroblasts and thus may represent an effective therapeutic approach for the regulation of wound healing progression (Bainbridge, 2013; Sarkhail et al., 2020). Therefore, *J. montana* extracts and pure isolated compounds were evaluated to determine whether they have any effect on the production of several inflammatory cytokines. Our findings suggest that fractions from *J. montana* have varying degrees of *in vitro* anti-inflammatory effects on human skin fibroblasts by modulating the levels of IL-1β, IL-6 and IL-8 (Figures 3–5). Furthermore, the inhibition of inflammatory mediator levels was not due to an overall cytotoxic effect, as described in a previous study (Juszczak et al., 2021), except for high concentrations of **22** and the luteolin-rich fraction **JM4** with antiproliferative and cytotoxic potential, which may be related to the activation of caspases (Juszczak et al., 2021). Many well-known anti-inflammatory molecules may inhibit the activity of the enzymes belonging to caspase-family. For example, numerous non-steroidal anti-inflammatory drugs (NSAIDs), such as propionic acid derivatives (tenbufen, indoprofen, and iaprofenic acid), acetic acid derivatives (ketorolac, felbinac and tolmetin), as well as others such as ebselen and flunixin are potent multi-caspase inhibitors. Furthermore, it seems that the inhibition occurs at physiologically relevant concentrations *in vitro* and *in vivo*, as well as that it is cyclooxygenase-independent (Smith et al., 2017). *In vitro* and docking studies show that other anti-inflammatory drugs, such as colchicine, and the corticosteroid drugs, dexamethasone and methylprednisolone, also supress the caspase-1 activity (Ben-Chetrit, 2018; Caruso et al., 2022). Numerous natural phenols such as anthranoids (chrysophanol) (Kim et al., 2010), and flavonoids (icariin and taxifolin) (Zu et al., 2019; Zhan et al., 2021) may also influence caspase activity. Thus, the study of small molecules as caspase inhibitors represents a developing area of research on new anti-inflammatory drugs. Previous studies have reported the anti-inflammatory effects of luteolin and its derivatives (López-Lázaro, 2009; Aziz et al., 2018). Karatoprak and coauthors suggested that the inhibitory effect of *A. mollis* extracts on TNF-α secretion, which in addition to initiating and propagating inflammation upregulates the already mentioned pro-inflammatory cytokines, is linked to the presence of luteolin 7-*O*-glucoside (Karatoprak et al., 2017). Furthermore, Ustuner and coauthors also attributed the anti-inflammatory effect of the *T. sipyleus* extracts to the presence of luteolin-7-*O*-glucoside (Ustuner et al., 2019). In an *in silico* study, the transcription factors Src, MAPK pathway and SOCS3 were found to be the main targets of the anti-inflammatory effect of luteolin 7-*O*-glucoside (Aziz et al., 2018). Wedler and coauthors suggested that a luteolin derivative probably responsible for the action of *Phyllostachys edulis* extract exerts moderate anti-inflammatory activity by inhibiting TNF-α-induced production of the proinflammatory cytokine IL-6 and the chemokine IL-8 in an in vitro model of HaCaT cells (Wedler et al., 2014). Moreover, our data on the anti-inflammatory activity of **22** and the **JM4** fraction are in good agreement with data from *in vitro* and *in vivo* studies during which inhibition of TNF-α and IL-6 secretion was observed (Yang et al., 2018) and from clinical studies reporting that luteolin has excellent therapeutic activity against inflammation-related disorders (Ustuner et al., 2019).

In conclusion, extracts from the aerial parts of *J. montana* have been shown to have high antioxidant activity, promote viability and accelerate the migration of fibroblasts. These mechanisms focus on multiple phases of the dynamic wound healing process, making them, along with the described anti-inflammatory, anti-elastase and stimulating collagen synthesis activities, the main factors in wound healing. Hence, the aboveground parts of *J. montana*, rich in flavonoid compounds, may be potentially useful for topical therapeutic application to stimulate the wound healing process.

## Data Availability

The original contributions presented in the study are included in the article/Supplementary Material, further inquiries can be directed to the corresponding author.
