# Supplementary material for: Wound Healing Properties of Jasione montana Extracts and Their Main Secondary Metabolites
Source: Front Pharmacol. 2022 May 10;13:894233. doi: 10.3389/fphar.2022.894233 (PMC9127232; doi:10.3389/fphar.2022.894233)
Supplement: Supplementary file 1 [file DataSheet1.docx]

***Supplementary Material***

**Wound healing properties of *Jasione montana* extracts and their main secondary metabolites**

**Aleksandra Maria Juszczak^1^, Katarzyna Jakimiuk^1^, Robert Czarnomysy^2^, Jakub Władysław Strawa^1^, Marijana Zovko Končić^3^, Krzysztof Bielawski^2^, and Michał Tomczyk^1*^**

^1^ Department of Pharmacognosy, Faculty of Pharmacy with the Division of Laboratory Medicine, Medical University of Białystok, ul. Mickiewicza 2a, 15-230 Białystok, Poland

^2^ Department of Synthesis and Technology of Drugs, Faculty of Pharmacy with the Division of Laboratory Medicine, Medical University of Białystok, ul. Kilińskiego 1, 15-089 Białystok, Poland

^3^ Department of Pharmacognosy, Faculty of Pharmacy and Biochemistry, University of Zagreb, Marulićev trg 20/II, 10000 Zagreb, Croatia

*** Correspondence:**

Michał Tomczyk

michal.tomczyk@umb.edu.pl

**Keywords: *Jasione montana*, Campanulaceae, flavonoids, luteolin derivatives, fibroblasts, wound healing, antioxidant, enzyme inhibition**

# Plant material

The aboveground parts of *J. montana* were collected from plants occurring in their natural habitat within the area of Puszcza Knyszyńska (N53°15’20.9”; E23°25’41.5”) in the region of Supraśl (Podlasie Province, Poland) within the period of June–August 2017 and 2018. Samples of the collected plant material were identified based on the scientific botanical literature, available the Royal Botanic Gardens database (https://powo.science.kew.org/) and its morphological features by one of the authors (M.T.) (Rutkowski, 2006). A plant voucher specimen (JM-15029) has been deposited in the Herbarium of the Department of Pharmacognosy at the Medical University of Białystok, Poland.

# Chemicals

2,2-diphenyl-1-picrylhydrazyl (DPPH) (CAS1898-66-4), methanol (MeOH) (CAS67-56-1), trolox (CAS53188-07-1), Ferric Reducing Antioxidant Power Assay Kit (MAK369), Tris buffer (CAS77-86-1), elastase from porcine pancreas type I (PPE) (CAS39445-21-1), quercetin (CAS 849061-97-8), dimethyl sulfoxide (DMSO) (CAS67-68-5), allantoin (CAS97-59-6), formic acid (HCOOH) (CAS64-18-6) were purchased from Sigma Aldrich Co. (St. Louis, MO, USA). N-Succ-Ala-Ala-Ala-p-nitroanilide (AAAPVN) (CAS52299-14-6) was purchased from Serva Electrophoresis (Heidelberg, Germany). The normal human dermal fibroblast cell line (PCS-201-012) was purchased from the American Type Culture Collection (ATCC; Manassas, VA, USA). Dulbecco’s minimal essential medium (DMEM), fetal bovine serum (FBS), phosphate-buffered saline (PBS), trypsin EDTA, glutamine, penicillin, and streptomycin were acquired from Corning (Corning, NY, USA). Stain Buffer was acquired from BD Pharmingen (San Diego, CA, USA). Anti-Collagen Type I (RABBIT) antibody fluorescein conjugated was purchased from the Rockland Immunochemicals, Inc. (Pottstown, PA, USA). Cytometric Bead Array (CBA) Human Inflammatory Cytokines Kit was obtained from BD Biosciences (San Jose, CA, USA). Luteolin (CAS491703) and luteolin 7-*O*-glucoside (CAS5373115) used as standards for quantification analysis were purchased from Chemat (Gdańsk, Poland). Acetonitrile (MeCN) (CAS75-05-08) was purchased from Fisher Chemical (Thermo Fisher Scientific, Leicestershire, UK). Water (UPW) type I (18.2 MΩxcm resistivities at 25℃ ) was obtained using a deionizer POLWATER DL3-100 (Labopol, Kraków, Poland).

# HPLC–PDA condition

To establish the quantitative content of the main compounds of **JM1**–**JM6**, HPLC–PDA analysis was performed on a 1260 Infinity chromatography system (Agilent Technologies, Santa Clara, CA, USA). The analysis was performed using a Kinetex XB-C18 column (150 × 2.1 mm, 1.7 µm; Phenomenex, Torrance, CA, USA) secured by pre-column and thermostated at 25°C. Compounds were eluted at flow rate 0.1 mL/min with UPW and MeCN acidified with HCOOH (0.1%) in 3 steps of a linear gradient (22 min - 28% B, 35 min - 75% B, 45 min - 95% B) after 1.5 min of initial conditions (5% B). The analysis was completed with a 3 min cleaning of the column (95% B), and a 6 min equilibration before next injection. Chromatogram UV were recorded at 348 nm wavelengths matching the value of the maximum absorption of the selected standards.

## Preparation of calibration curves

Exactly weighed standard substances were dissolved, filtered through a 0.2 µm PVDF syringe filter to give a final concentration of 1 mg/ml (*m*/*v*). In the next step, stock solutions were used to create a mixture of patterns. The working range of the curves from 6 concentration levels was then obtained by the multiple dilution method with the mobile phase. Then, were immediately used to determine the standard curve.

## Preparation of samples solution

Accurately weighed samples were dissolved, then centrifuged and filtered through a 0.2 µm PVDF syringe filter into a volumetric flask to give a final 5 mg/mL and 2 mg/mL concentration of extracts and fractions, respectively.

# Method Validation

### Selectivity

Three solutions were made for tested extracts to exclude the occurrence of co-elution. Analysis with the use of a PDA detector at three selected wavelengths was used. The analysis of the obtained UV chromatograms made it possible to exclude the possibility of elution of the compounds at the same time.

### Linearity

Standard solutions at six concentration levels were analyzed in triplicate. Calibration curves were generated using linear regression on the plots of peak area of each standard versus injected content to the column. Linear regression parameters for the standard curve were determined using ANOVA. Statistical significance has been confirmed. Calculations were made using MS Excel 2019 with a Data Analysis add-on.

### Limits of Detection (LOD) and Quantification (LOQ)

In accordance with the International Council for Harmonisation of Technical Requirements for Pharmaceuticals for Human Use (ICH) recommendations the detection limit and limit of quantification based on the standard deviation (σ) of the response and the slope (S). Repeating for ICH, we assumed LOD = 3.3σ/S and LOQ = 10σ/S. The parameters are summarized in Supplementary Table S1.

**Supplementary Table S1.** Regression data, the limit of detection (LOD) and limit of quantification (LOQ), accuracy and precision obtained during HPLC–PDA method validation.

| **Parameter** | **luteolin 7-*O-*glucoside (12)** | **luteolin (22)** |
| --- | --- | --- |
| Linear range [µg/mL] | 25–1000 | 5–500 |
| R^2^ (N=6) | 0.9999 | 0.9999 |
| Regression equation ^a^ | y = 22851x+106.69 | y = 36173x-34.602 |
| LOD [µg/mL] | 19.4 | 4.6 |
| LOQ [µg/mL] | 59 | 14 |
| Accuracy [%] | 101.09±3.23 | 101.23±2.93 |
| Intraday precision (%CV) (N=6) | 0.58 | 0.93 |
| Interday precision (%CV) (N=9) | 0.88 | 1.26 |

^a^ the value for y corresponds to the peak area and x to the concentration, respectively


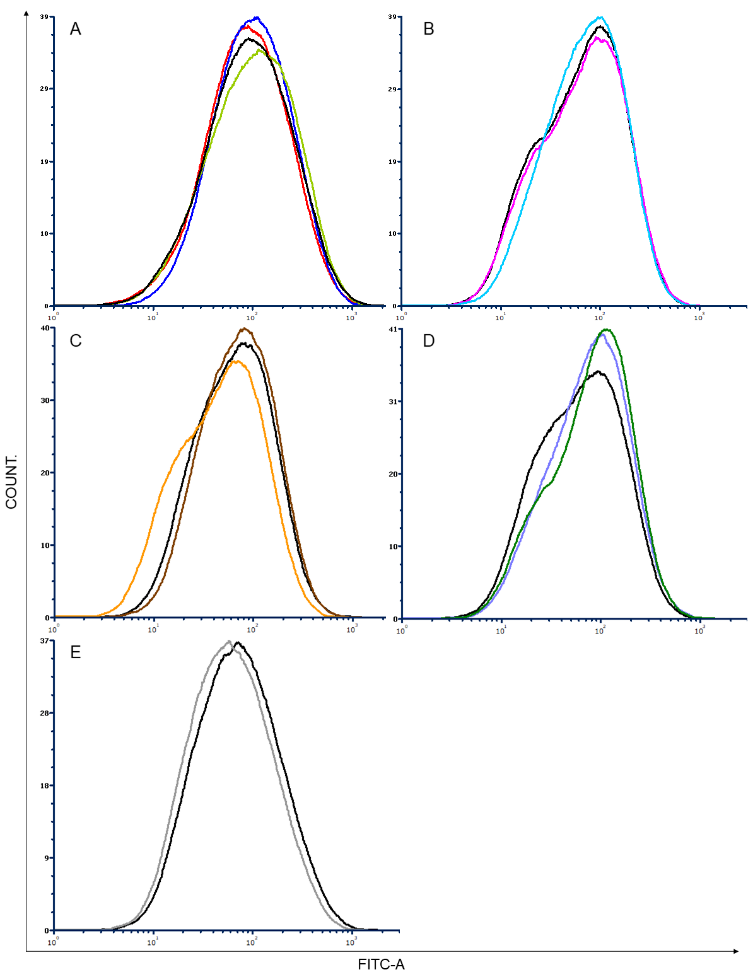


**Supplementary Figure S1.** Expression of Collagen Type 1 antibody in fibroblast cells after 24 h incubation
with *J. montana* extracts (**JM1**–**JM6**) and their main compounds (**9**, **12**, **22**) (10–300 μg/mL) analysis by FCS Express 7 software (DeNovo Software, Pasadena, CA, USA). Representative histograms were derived from flow cytometric analysis of 10.000 cells and show control cells (black line), (A) **JM1**-treated cells (red line), **JM2**-treated cells (blue line) and **JM3**-treated cells (light green line), (B) **JM4**-treated cells (pink line) and **JM5**-treated cells (light blue line), (C) **JM6**-treated cells (brown line) and **22**-treated cells (orange line), (D) **12**-treated cells (purple line) and **9**-treated cells (dark green line) and (E) cells treated with positive control allantoin (gray line).

# References

Rutkowski, L. (2006). *Klucz do oznaczania roślin naczyniowych Polski niżowej*. Warsaw: Wydawnictwo Naukowe PWN.
